# Regional Cerebral Oximetry as an Indicator of Acute Brain Injury in Adults Undergoing Veno-Arterial Extracorporeal Membrane Oxygenation–A Prospective Pilot Study

**DOI:** 10.3389/fneur.2018.00993

**Published:** 2018-11-23

**Authors:** Imad Khan, Mehboob Rehan, Gunjan Parikh, Christopher Zammit, Neeraj Badjatia, Daniel Herr, Zachary Kon, Charles Hogue, Michael Mazzeffi

**Affiliations:** ^1^Division of Neurocritical Care, Department of Neurology, University of Rochester School of Medicine, Rochester, NY, United States; ^2^Department of Medicine, Eastern Idaho Regional Medical Center, Idaho Falls, ID, United States; ^3^Section of Neurocritical Care and Emergency Neurology, Department of Neurology, University of Maryland School of Medicine, Baltimore, MD, United States; ^4^Division of Critical Care Medicine, Department of Medicine, University of Maryland School of Medicine, Baltimore, MD, United States; ^5^Division of Cardiothoracic Surgery, Department of Surgery, University of Maryland School of Medicine, Baltimore, MD, United States; ^6^Department of Anesthesiology, Northwestern University Feinberg School of Medicine, Chicago, IL, United States; ^7^Department of Anesthesiology, University of Maryland School of Medicine, Baltimore, MD, United States

**Keywords:** NIRS (near infrared reflectance spectroscopy), cerebral oximetry, ECMO (extracorporeal membrane oxygenation), acute brain injury, neurological outcome, adults'

## Abstract

**Background:** Regional cerebral oxygen saturation (rScO2) measured by near-infrared spectroscopy (NIRS) can be used to monitor brain oxygenation in extracorporeal membrane oxygenation (ECMO). ECMO patients that develop acute brain injuries (ABIs) are observed to have worse outcomes. We evaluated the association between rScO2 and ABI in venoarterial (VA) ECMO patients.

**Methods:** We retrospectively reviewed prospectively-collected NIRS data from patients undergoing VA ECMO from April 2016 to October 2016. Baseline demographics, ECMO and clinical characteristics, cerebral oximetry data, neuroradiographic images, and functional outcomes were reviewed for each patient. rScO2 desaturations were defined as a >25% decline from baseline or an absolute value < 40% and quantified by frequency, duration, and area under the curve per hour of NIRS monitoring (AUC rate, rScO2^*^min/h). The primary outcome was ABI, defined as abnormalities noted on brain computerized tomography (CT) or magnetic resonance imaging (MRI) obtained during or after ECMO therapy.

**Results:** Eighteen of Twenty patients who underwent NIRS monitoring while on VA ECMO were included in analysis. Eleven patients (61%) experienced rScO2 desaturations. Patients with desaturations were more frequently female (73 vs. 14%, *p* = 0.05), had acute liver dysfunction (64 vs. 14%, *p* = 0.05), and higher peak total bilirubin (5.2 mg/dL vs. 1.4 mg/dL, *p* = 0.02). Six (33%) patients exhibited ABI, and had lower pre-ECMO Glasgow Coma Scale (GCS) scores (5 vs. 10, *p* = 0.03) and higher peak total bilirubin levels (7.3 vs. 1.4, *p* = 0.009). All ABI patients experienced rScO2 desaturation while 42% of patients without ABI experienced desaturation (*p* = 0.04). ABI patients had higher AUC rates than non-ABI patients (right hemisphere: 5.7 vs. 0, *p* = 0.01, left hemisphere: 119 vs. 0, *p* = 0.06), more desaturation events (13 vs. 0, *p* = 0.05), longer desaturation duration (2:33 vs. 0, *p* = 0.002), and more severe desaturation events with rScO2 < 40 (9 vs. 0, *p* = 0.05). Patients with ABI had lower GCS scores (post-ECMO initiation) before care withdrawal or discharge than those without ABI (10 vs. 15, *p* = 0.02).

**Conclusions:** The presence and burden of cerebral desaturations noted on NIRS cerebral oximetry are associated with secondary neurologic injury in adults undergoing VA ECMO.

## Introduction

Extracorporeal membrane oxygenation (ECMO) is increasingly being used in adults with cardiac failure and cardiac arrest (1). While survival is improving, between 7 and 15% of adults undergoing ECMO are found to have potentially devastating and often debilitating neurological complications such as infarction, hemorrhage, and seizures (2, 3). In both veno-arterial (VA) and veno-venous (VV) ECMO patients, mortality is higher in patients with neurological injury than in patients without (1, 2).

Near infrared spectroscopy (NIRS) is a non-invasive tool that can be used to monitor regional saturation of cerebral oxygen (rScO2) and holds promise as a real-time bedside target for goal-directed ECMO therapy. This technology can demonstrate improvement in brain oxygenation in patients undergoing VV ECMO for acute respiratory distress syndrome (ARDS) (4), and has been used to titrate VA ECMO therapy in a patient with cardiac arrest (5). NIRS is also used intraoperatively to monitor patients undergoing cardiac surgery as desaturations in rScO2 can indicate post-surgical cognitive decline and stroke (6–8). However, it is unclear whether rScO2 desaturations associate with acute brain injury (ABI) in adults undergoing ECMO for acute cardiac failure. In this prospective cohort study, we sought to evaluate whether rScO2 desaturations were associated with radiographic brain injury in adult VA ECMO patients.

## Materials and methods

### Study population

We conducted a retrospective analysis of prospectively collected data of consecutively-admitted adult patients at our medical center who received rScO2 monitoring during VA ECMO between April 1, 2016 and October 31, 2016. rScO2 monitoring was performed on all adult patients undergoing VA ECMO during that time period as an evaluation of a new NIRS device in the cardiothoracic ICU. Per institutional standard of care, patients with known pre-morbid neurologic injury or poor baseline level of function were not cannulated for ECMO. Patients who were on ECMO for less than 24 h were excluded because they underwent ECMO as a method of weaning from cardiopulmonary bypass after elective cardiac surgery, not for emergent cardiac failure typical of longer ECMO patients. The Institutional Review Board approved the study and informed consent was waived.

### Patient and ECMO data

Baseline demographic data and ECMO data were recorded prior to developing outcome of interest including indication for ECMO, cannulation type (peripheral vs. central), initial sweep gas flow, and initial ECMO blood flow. Comorbidities on admission were recorded. Lab values on admission, daily during admission, and during desaturations were recorded. The following organ failures during ECMO were recorded: acute renal failure requiring continuous renal replacement therapy (CRRT) and new onset liver dysfunction defined by *de novo* elevation of international normalized ratio (INR) >1.5 with transaminases >3 × the upper limit of normal. The Glasgow Coma Scale (GCS) was used to define baseline neurologic function and was obtained from nursing documentation prior to the initiation of ECMO.

### ECMO management

Cardiothoracic surgeons performed ECMO cannulations in the intensive care unit or operating room. Either peripheral or central cannulation was used depending on surgeon preference. Initial sweep gas flows were set by the cannulating surgeon and were titrated according to the patient's arterial pH and partial pressure of carbon dioxide (pCO2). Initial ECMO blood flows were set by the cannulating surgeon and were adjusted to maintain goal MAP >65 mm Hg and cardiac index greater than 2 L/min/m^2^. Patient temperature was managed using a heat exchanger attached to the ECMO circuit. Patient temperatures were maintained between 36 and 38C°. In patients who were placed on ECMO after cardiac arrest, temperature was strictly maintained at 36 C° for 24 h per institutional standard of care, after which they were rewarmed to 37 C° by 0.1 C°/h.

### NIRS monitoring

NIRS monitoring was performed using the Covidien INVOS 5100c Cerebral Oximeter (Medtronic, Minneapolis, MN, USA). Monitoring was started within 24 h of cannulation and continued until ECMO decannulation. Sensors were attached to both sides of the forehead, each with one light-emitting diode emitting near-infrared wavelength light at 730 and 810 nm and two detectors (9). Sensors were routinely replaced every 5 days or if proper adhesion was lost. rScO2 was displayed on the INVOS monitor every 3 s, automatically recorded on a secure USB drive attached to it, and then uploaded to a computer for analysis. Patient care teams were blinded to rScO2 data as the medical center was conducting a pilot study of the device's feasibility during the period of data collection.

rScO2 values were analyzed using the INVOS Analytics Tool (Medtronic, Minneapolis, MN, USA) and visual inspection by two of the authors (IK and MR). Initial values were labeled as the baseline, and desaturations were defined as a drop in rScO2 > 25% below baseline or an absolute rScO2 < 40%, based on a previous study that examined cerebral oximetry in adults undergoing ECMO (10). The start time of each desaturation was recorded at the beginning of a downward trend in rScO2 and the time point at which rScO2 began steadily increasing was labeled as the end time. An area under the curve (AUC, rScO2^*^min) value was automatically calculated by the Analytics Tool via a proprietary formula in order to quantify the degree of desaturation. The threshold below which rScO2 would be considered “under the curve” is set by the user in the Analytics Tool, and we set this threshold to be at 25% below the baseline rScO2. Each patient's total AUC was divided by the number of hours of rScO2 monitoring he/she underwent to devise an hourly AUC rate (rScO2^*^min/h), in order to account for differences in the duration of monitoring. An AUC, and AUC rate, was documented for both the right and left sensors and labeled correspondingly.

### Study outcomes

The study's primary outcome was acute brain injury (ABI) seen on radiographic imaging. Neuroimaging was obtained at the discretion of the medical team if there was clinical suspicion of brain injury. Brain injury was evaluated by review of available computed tomography (CT) scan and/or magnetic resonance imaging (MRI) scans that were conducted during or after ECMO. Two study authors independently adjudicated all injuries (IK and MR). As noted above, desaturations were never used as the sole indication for neuroimaging as they were not implicated in clinical decision-making. Patients who did not receive neuroimaging were added to the non-ABI group for statistical analysis. Secondary outcomes included good functional status at the time of discharge, defined by Cerebral Performance Category (CPC) 1 or 2 (11). This score was calculated by review of discharge, physical, and occupational therapy notes. The CPC score was utilized because of its prior validation in cardiac arrest populations, which was the most common indication for ECMO in our cohort. We also recorded the last nurse-documented GCS score prior to the patient's discharge, death, or withdrawal of care as a final assessment of the patient's neurologic status. This assessment could have been made during ECMO therapy or after its discontinuation if the patient survived to that point.

### Statistical analysis

Statistical analysis was performed using SPSS (Version 25.0, IBM Corp, Armonk, NY). Normality of variables were assessed using the Shapiro-Wilk test. Demographic, clinical, and rScO2 characteristics of patients were compared using Student's *T*-test (for normally distributed continuous variables), the Wilcoxon Rank Sum test (for non-normally distributed continuous variables), or Chi-Squared Test (for categorical variables). Characteristics were summarized as the mean ± 2 standard deviations, median (1st, 3rd quartiles), or n (%) depending on variable type and normality of distribution. Study outcomes were compared between patient groups using the Chi-Squared test or the Wilcoxon Rank Sum test. A *p*-value ≤ 0.05 was used to exclude the null hypothesis.

## Results

Twenty consecutive VA ECMO patients underwent rScO2 monitoring between April 1, 2016 and October 31, 2016. Two patients were excluded from the analysis because of poor data quality (missing data for >50% of ECMO time, due to disconnection of sensor pads), leaving 18 patients in the final analysis. High-quality data was available for >75% of the monitoring duration for all patients included in the analysis. Time periods with absent recorded rScO2 values were not included in the analysis. VA ECMO was performed for extracorporeal cardiopulmonary resuscitation (ECPR) for cardiac arrest in 9 patients, post-cardiotomy shock in 1 patient, massive or submassive pulmonary embolism in 3 patients, and acute cardiogenic shock from other causes in 5 patients. Other causes of cardiogenic shock included non-ischemic cardiomyopathy (1), ventricular septal defect (1), severe mitral stenosis (1), and ST-elevation myocardial infarction (2). Sixteen patients were cannulated peripherally and 2 patients were cannulated centrally.

**Figure 1:**
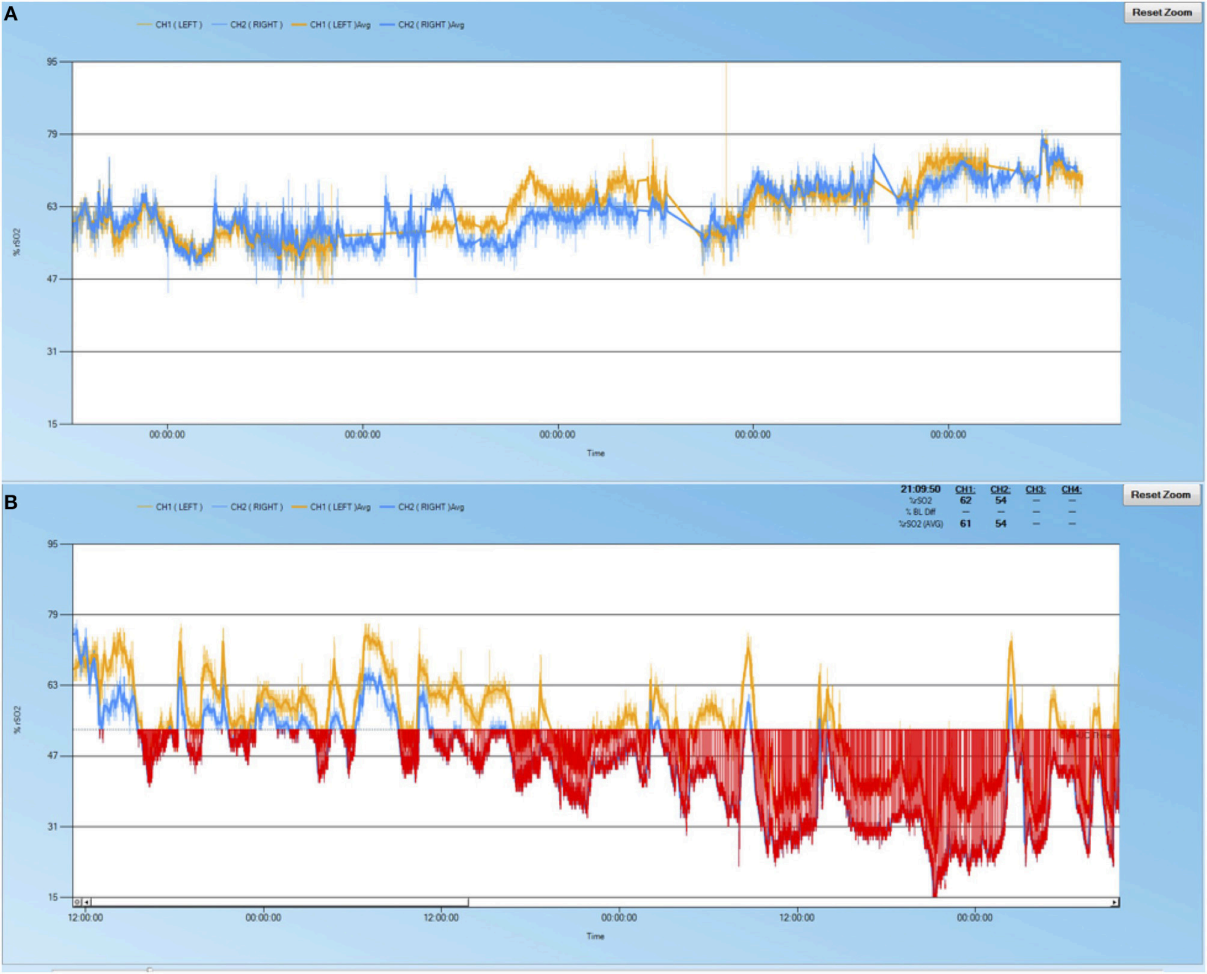
Sample cerebral oximetry time course of patients. **(A)** 69 year-old male with pulmonary embolus, no acute liver injury, no rScO2 desaturations, normal neuroimaging, and survived to discharge. **(B)** 64 year-old male with cardiogenic shock, acute liver injury, significant rScO2 burden (red shaded area), watershed infarctions noted on neuroimaging, did not survive to discharge.

rScO2 desaturations occurred in 11 of 18 patients (61%) (Table 1). Examples of cerebral oximetry graphs of patients with and without desaturations are displayed in Figure 1. Patients with rScO2 desaturations were more often female (73 vs. 14%, *p* = 0.05), had acute liver dysfunction (64 vs. 14%, *p* = 0.05), and had higher peak total bilirubin (5.2 mg/dL vs. 1.4 mg/dL, *p* = 0.02). ABI occurred in 6 patients (33%) (Table 1). These patients had lower pre-ECMO GCS scores (5 vs. 10, *p* = 0.03) and higher peak total bilirubin levels (7.3 vs. 1.4, *p* = 0.009).

**Table 1:** Patient characteristics^a^.

**Characteristic**	**Desaturations (*n* = 11)**	**No desaturations (*n* = 7)**	**ABI (*n* = 6)**	**No ABI (*n* = 12)**
Age	61 (24, 69)	67 (56, 73)	63 (57, 69)	57 (34, 71)
**SEX**				
Male	3 (27%)	6 (85%)	2 (33%)	7 (58%)
Female	8 (73%)	1 (14%)^*^	4 (67%)	5 (42%)
Pre-ECMO GCS^b^	7.5 (3–15)	10 (3–15)	5 (3–10)	10 (3–15)^*^
**INDICATION FOR ECMO**				
ECPR	4 (36%)	5 (71%)	2 (33%)	7 (58%)
Post-cardiotomy	1 (9%)	0	1 (17%)	0
PE^c^	2 (18%)	1 (14%)	0	3 (25%)
Cardiogenic shock	4 (36%)	1 (14%)	3 (50%)	2 (17%)
**COMORBIDITIES**				
Hypertension	4 (36%)	5 (71%)	3 (50%)	6 (50%)
Diabetes	3 (27%)	2 (29%)	1 (17%)	4 (33%)
Liver dysfunction	7 (64%)	1 (14%)^*^	4 (67%)	4 (33%)
Baseline lactate^f^	4.1 (2.7, 9.3)	2.6 (1.7, 9.2)	4.1 (3.2, 9.6)	2.7 (2, 9.2)
Baseline creatinine^g^	1.7 ± 0.7	1.5 ± 0.8	1.8 (1.4, 2.4)	1.1 (0.9, 2.5)
Peak total bilirubin^g^	5.2 (1.8, 10.4)	1.4 (1.3, 1.5)^*^	7.3 (2.9, 11.6)	1.4 (1.3, 2.3)^*^
Baseline EF^h^	20 (10, 50)	55 (15, 75)	40 (20, 55)	23 (15, 60)
CRRT^d^	5 (45%)	3 (43%)	4 (67%)	4 (33%)
Hemorrhage	2 (18%)	2 (29%)	2 (33%)	2 (17%)
Blood transfusions^e^	5 (0–11)	11 (5–13)	5 (0–11)	7 (0–13)
Duration of ECMO (days)	9 (6, 11)	8 (4, 14)	9 (6, 13)	9 (5, 11)
**CANNULATION TYPE**				
Peripheral	10 (91%)	6 (86%)	5 (83%)	11 (92%)
Central	1 (9%)	1 (14%)	1 (17%)	1 (8%)
Initial sweep gas flow^i^	5.5 ± 2	4.6 ± 2.3	6.3 ± 2.3	4.6 ± 1.9
Initial blood flow^i^	4.4 ± 0.5	4.4 ± 1.1	4.4 ± 0.5	4.4 ± 0.9

a Normally-distributed variables are reported as mean ± SD, non-normally distributed variables reported as median (1st, 3rd quartiles);

b GCS, Glasgow Coma Scale, median (range);

c PE, pulmonary embolus;

d CRRT, continuous renal replacement therapy;

e Total units during ECMO, median (range);

f in mmol/dL;

g in mg/dL;

h ejection fraction, in %;

i in liters/min;

* p ≤ 0.05

Survivors had lower baseline rScO2 values than non-survivors (right: 57 ± 9 vs. 65 ± 6, *p* = 0.04; left: 57 ± 12 vs. 68 ± 8, *p* = 0.05) and had higher average hemoglobin values at the time of cerebral desaturation (10.9 vs. 8.5 mg/dL, *p* = 0.02) (Table 2). There was no significant difference in the number of desaturation events or area under the desaturation curve between survivors and non-survivors. All patients with ABI experienced a desaturation, while 42% of patients without ABI experienced a desaturation (*p* = 0.04) (Table 2). Both unilateral (4 vs. 0, *p* = 0.02) and bilateral (10 vs. 0, *p* = 0.05) desaturation patterns were seen more in ABI patients. ABI patients had significantly more desaturation events (13 vs. 0, *p* = 0.05), higher right AUC rates (5.7 vs. 0, *p* = 0.01), more severe desaturation events with rScO2 < 40 (9 vs. 0, *p* = 0.05), and had longer durations of desaturation (2:33 vs. 0, *p* = 0.002). The left AUC rate in patients with ABI trended toward significantly higher values than in those without (119 vs. 0, *p* = 0.06).

**Table 2:** Regional brain oxygenation values^a^.

**Characteristic**	**Non-survivors (*n* = 7)**	**Survivors (*n* = 11)**	**ABI (*n* = 6)**	**No ABI (*n* = 12)**
**BASELINE RSCO2**^b^ **(%)**				
Left	68 ± 8	57 ± 12*	64 ± 11	60 ± 12
Right	65 ± 6	57 ± 9*	64 ± 9	58 ± 9
Experienced desaturation event	5 (71%)	6 (55%)	6 (100%)	5 (42%)*
Number of desaturation events	8 (0, 29)	4 (0, 15)	13 (8, 19)	0 (0, 14)*
**AVERAGE AUC RATE**^c^				
Left	0.5 (0, 236)	0.8 (0, 75.9)	119 (0.5, 327)	0 (0, 4.7)
Right	3.2 (0, 8.1)	0.6 (0, 2)	5.7 (0.7, 113)	0 (0, 1.4)*
No. events rScO2^b^ < 40	2 (0, 7)	0 (0, 10)	9 (2, 15)	0 (0, 4)*
Duration per patient^d^	0:47 (0, 3:51)	0:24 (0, 2:03)	2:33 (0:54, 3:51)	0 (0, 0:38)*
Average minimum rScO2^b^ (%)	45 ± 11	41 ± 12	38 ± 10	45 ± 12
**DESATURATION PATTERN**				
No. of unilateral	2 (0, 5)	0 (0, 0)	4 (0, 6)	0 (0, 0)*
No. of bilateral	6 (0, 27)	4 (0, 10)	10 (6, 11)	0 (0, 11)*
Average Hb at time of desaturation^e^	8.5 (8.3, 8.4)	10.9 (8.8, 10)*	8.6 (8.4, 9.1)	9.7 (8.9, 9.8)
Events after blood flow change	1 (0, 2)	0 (0, 0)	0 (0, 1)	0 (0, 0)
Events after sweep change	1 (0, 3)	1 (0, 1)	1 (0, 3)	0 (0, 1)

a Normally-distributed variables reported as mean ± SD, non-normally distributed variables reported as median (1st, 3rd quartiles);

b rScO2, regional saturation of cerebral oxygen;

c AUC, area under the curve, rate in rScO2*min/h;

d in hours:minutes;

e Hb, hemoglobin, in mg/dL;

* *p ≤ 0.05*.

Fourteen of the 18 patients had neuroimaging (78%): 2 had MRIs after ECMO and 12 had CT scans during or after ECMO. Neuroimaging findings are detailed in Table 3. Five patients (35%) with neuroimaging had brain infarctions, consisting of 2 watershed infarctions, 2 embolic infarctions, and one patient with both. One patient (17%) with recent cardiac arrest had diffuse cerebral edema on CT scan. Microhemorrhages were noted on 2 (14%) images, both of which were MRI's. Four of the Eighteen patients (22%) did not clinically warrant neuroimaging. These patients were younger (36 vs. 60 years old, *p* = 0.02) and had higher pre-ECMO GCS scores (11 vs. 6, *p* = 0.05). The one patient who died before discharge and never received neuroimaging had a history of right ventricular failure, suffered a cardiac arrest warranting ECPR, and then developed septic shock with fungemia.

**Table 3:** Characteristics of groups with and without neuroimaging.

**Characteristic**	**Obtained imaging (*n* = 14)**	**Did not obtain imaging (*n* = 4)**	***p***
Imaging result		N/A	N/A
Normal	8 (57%)	
Watershed infarction	3 (21%)	
Embolic infarction	3 (21%)	
Microhemorrhage	2 (14%)	
Diffuse cerebral edema	1 (7%)	
Age (years)	60 ± 14	36 ± 27	0.02
Pre-ECMO GCS^b^	6 (3–15)	11 (9–15)	0.05
Indication for ECMO			1
ECPR	7 (50%)	2 (50%)
Non-ECPR	7 (50%)	2 (50%)
Duration of ECMO (days)	8 (6, 13)	10 (7, 11)	0.9
Experienced desaturation	7 (50 %)	4 (100%)	0.1
**BASELINE rScO2**^c^ **(%)**		
Left	60 ± 12	68 ± 12	0.3
Right	59 ± 9	62 ± 8	0.6
Last recorded GCS	14 (4–15)	15 (13–15)	0.07
**FUNCTIONAL OUTCOME**		
Good (CPC 1-2)^d^	8 (57%)	3 (75%)	0.6
Died before discharge	6 (43%)	1 (25%)	0.6

a Normally-distributed variables are reported as mean ± SD, non-normally distributed variables reported as median (1st, 3rd quartiles);

b GCS, Glasgow Coma Scale, median (range);

c rScO2, regional saturation of cerebral oxygen;

d *CPC, cerebral performance category*.

Acute brain injury was noted in 6 of the 18 patients (33%) and all 6 (100%) experienced rScO2 desaturations (Table 4). No significant differences were found in neurologic function at discharge, in-hospital death, or last recorded GCS scores between patients with or without desaturations (Table 4). All patients who died had life sustaining treatment withdrawn by their family due to poor prognosis. Non-survivors had lower median GCS scores prior to withdrawal of care compared to survivors at the time of discharge (4 vs. 15, *p* < 0.001). Patients with ABI also had lower last recorded GCS scores than those without ABI (10 vs. 15, *p* = 0.02).

**Table 4:** Patient outcomes^a^.

**Characteristic**	**Desaturations (*n* = 11)**	**No desaturations (*n* = 7)**	***p***	**ABI (*n* = 6)**	**No ABI (*n* = 12)**	***p***
Acute brain injury			0.04	N/A	N/A
Absent	5 (45%)	7 (100%)			
Present	6 (55%)	0 (0%)			
Functional outcome					
Good (CPC 1-2)^b^	6 (55%)	5 (71%)	0.6	2 (33%)	9 (75%)	0.1
Died before discharge	5 (45%)	2 (29%)	0.6	4 (67%)	3 (25%)	0.1
Last recorded GCS^c^	13 (3–15)	15 (3–15)	0.5	10 (3–14)	15 (3–15)	0.02

a All numbers reported as n (%) or median (1st, 3rd quartile);

b CPC, cerebral performance category;

c *GCS, Glasgow Coma Scale, median (range)*.

## Discussion

In our single-center prospective cohort study of VA ECMO patients, we found that rScO2 desaturations were frequent, occurring in 11 (61%) of 18 patients. Six patients (33%) had confirmed brain injury. All patients with ABI experienced rScO2 desaturations during ECMO, and had more numerous, longer, and more severe events than those without ABI.

Establishing a method of monitoring the brain during ECMO is of key importance because the clinical exam, which is the gold standard for evaluating brain function, is often obviated by the requirement for sedation and/or neuromuscular blockade. Previous studies have evaluated cerebral oximetry with NIRS in ECMO patients, but most were in pediatric or neonatal populations (12–16). Three studies found that NIRS can detect cerebral hypoxia during carotid artery ligation in neonates (12, 13, 15). Another pediatric study used NIRS to demonstrate that autoregulation is impaired during periods of fluctuating ECMO blood flow (14). As NIRS allows for the continuous, non-invasive, portable monitoring of brain oxygen content, it is well-suited for monitoring for brain injury in the adult ECMO population (17). NIRS oximetry has also been shown to correlate closely with jugular bulb oximetry in pediatric patients undergoing cardiopulmonary bypass, a well-described monitoring technique in a population physiologically similar to VA ECMO (18, 19).

Patients who suffered acute brain injury appeared to have an overall higher burden of rScO2 desaturations than those without acute brain injury. This finding corroborates the two previous studies performed using NIRS to monitor rScO2 in the adult ECMO population (10, 20). In these studies, between 94 and 100% of the patients with confirmed neurologic injury experienced desaturations in rScO2, making cerebral oximetry with NIRS a highly sensitive screening tool. Our study differed from the prior two studies in that rScO2 values were blinded to patient care teams as the device was being tested in a pilot study. This allowed us to observe the natural history of cerebral oxygen saturation, and significant differences in desaturation duration and burden (AUC/h) became apparent between ABI and non-ABI groups.

The ABI group was noted to have a significantly higher AUC rate on the right hemisphere than the non-ABI group (Table 2). This may signify the presence of differential hypoxia, which is the partial perfusion of the brain with deoxygenated blood pumped from the patient's heart as it recovers and regains contractility while the lungs remain injured (21). This presents an intervenable moment, as possible treatment strategies to maintain the supply of oxygenated blood include increasing the ECMO circuit's flow rate, decreasing the heart's preload or inotropy, or introducing oxygen-rich blood via a second venous ECMO cannula (22). With the ability to monitor for differential hypoxia continuously at the bedside, NIRS may potentially provide a target for goal-directed ECMO therapy, which remains to be seen in future studies.

We found pre-ECMO GCS scores to be significantly lower in patients with ABI and lower in patients with desaturations, although this did not reach statistical significance (Table 1). This suggests the possibility that patients may have suffered ABI before the initiation of ECMO, which could confound the temporal relationship between desaturations and ABI. However, our study was not designed to determine a temporal relationship between desaturations and ABI, but merely an association between the two. Interestingly, although patients with ABI had lower pre-ECMO GCS scores, they did not appear to have lower baseline rScO2 values at the start of NIRS monitoring. Nevertheless, this group had a higher burden of desaturation. One possible explanation for this finding is that the ABI group may have had an underlying impairment of cerebral autoregulation making them prone to desaturation, although this remains to be elucidated in further studies.

A few baseline characteristics differentiated patients with desaturations vs. those without. First, female patients were more likely to experience desaturation (Table 1). This finding may be driven in part by sex differences in cerebral autoregulation (23, 24). Second, patients with desaturations had higher peak bilirubin levels and more often had acute liver dysfunction (Table 1). Patients with acute liver dysfunction can be at risk of cerebral hyper- or hypo-perfusion due to impaired cerebral autoregulation (25, 26). On the other hand, hyperbilirubinemia could falsely depress regional brain oxygenation values, as bilirubin can absorb near-infrared light (27). This confounding effect is challenged by the fact that bilirubin levels were also significantly elevated in the ABI group. The effect of acute liver dysfunction and hyperbilirubinemia on cerebral autoregulation in patients undergoing ECMO remains to be further described in future studies.

We found baseline rScO2 values to be higher in non-survivors than survivors in our study (Table 2), which was unexpected. This could be explained by the fact that patients with more severe brain injury, particularly anoxic injury, have lower cerebral oxygen consumption (28). Lower oxygen consumption increases venous oxygen saturation and subsequently could increase rScO2, which largely reflects the venous content of the brain (29). Cerebral ischemic preconditioning may offer another explanation for these findings, where the systemic ischemia experienced from cardiogenic shock may have lead to a vasodilatory state (30, 31). There were no other significant differences in rScO2 values between survivors and non-survivors including number of desaturation events and AUC, but our study was small and likely underpowered for these analyses.

In a recent retrospective review of an international ECMO registry, the survival rate for adults undergoing VA ECMO with radiographically-confirmed cerebral infarction or hemorrhage was 17.4 and 10.5%, respectively, vs. 57% for those without any neurologic injury (3). In our study, while the presence of desaturation was associated with radiographic brain injury, it did not correlate with survival and functional outcome. Our prospective pilot study was not adequately powered to determine a difference in these outcomes, and larger studies will be needed to determine if cerebral desaturation can independently predict functional outcome after ECMO.

We included the four patients who did not undergo neuroimaging into the non-ABI group because they did not clinically warrant neuroimaging. These patients had higher median pre-ECMO GCS scores and were significantly younger than those who received neuroimaging (Table 3). We did not find a significant difference in age between patients with or without ABI, or with or without desaturations (Table 1). Our study was not powered to be able to correlate age and occurrence of ABI, but it is notable that no age difference was noted in patients with or without acute cerebral complications in another recently published study examining cerebral desaturations in adults undergoing ECMO (20). However, the authors of that study did not describe the age of patients who underwent brain imaging vs. those without.

Our study has several limitations. First, it was a small prospective observational cohort study, which limits its generalizability and increases the possibility of Type 2 error. Our aim was to conduct a pilot study to evaluate the usefulness of NIRS technology to detect radiographic cerebral injury, and as such was not powered to detect differences in mortality or morbidity. Furthermore, as mentioned above, pre-ECMO brain injury could have confounded the association between cerebral desaturations and ABI, but this study was not intended to determine the causal relationship between the two. Second, rScO2 monitoring values can be skewed by elevated bilirubin levels, increased skull thickness, or superficial scalp deoxygenation, all of which could have affected our results (27, 32). Third, the system we used measures regional, not global cerebral oxygen saturation, and thus only a small portion of the cortex is evaluated. Thus, our device would not have detected deeper, subcortical pathology, or that of other regions of the cortex.

## Conclusions

In summary, our study data suggest that amongst patients undergoing VA ECMO, acute brain injury is associated with the frequency, duration, and burden of desaturations noted on NIRS cerebral oximetry. rScO2 is a promising biomarker for future goal-directed ECMO therapy studies that deserves further validation.

## Data availability statement

The raw data supporting the conclusions of this manuscript will be made available by the authors, without undue reservation, to any qualified researcher.

## Author contributions

IK and MM conceived the hypothesis and designed this study. IK and MR collected and reviewed data. IK was the primary author of the manuscript with input from all authors. IK performed statistical analysis. CH, GP, CZ, NB, DH, and ZK were involved in critical manuscript revisions. All authors read and approved the submitted version.

### Conflict of interest statement

The authors declare that the research was conducted in the absence of any commercial or financial relationships that could be construed as a potential conflict of interest.

## References

[1] Sauer CM Yuh DD Bonde P. Extracorporeal membrane oxygenation use has increased by 433% in adults in the United States from 2006 to 2011. ASAIO J. (2015) 61:31–6. 10.1097/MAT.0000000000000160 25303799

[2] Lorusso R Gelsomino S Parise O Di Mauro M Barili F Geskes G. Neurologic injury in adults supported with veno-venous extracorporeal membrane oxygenation for respiratory failure: findings from the extracorporeal life support organization database. Crit Care Med. (2017) 45:1389–97. 10.1097/CCM.0000000000002502 28538440

[3] Lorusso R Barili F Mauro MD Gelsomino S Parise O Rycus PT. In-Hospital neurologic complications in adult patients undergoing venoarterial extracorporeal membrane oxygenation: results from the extracorporeal life support organization registry. Crit Care Med. (2016) 44:e964–72. 10.1097/CCM.0000000000001865 27340754

[4] Kredel M Lubnow M Westermaier T Muller T Philipp A Lotz C. Cerebral tissue oxygenation during the initiation of venovenous ECMO. ASAIO J. (2014) 60:694–700. 10.1097/MAT.0000000000000128 25232765

[5] Taccone FS Fagnoul D Rondelet B Vincent JL de Backer D. Cerebral oximetry during extracorporeal cardiopulmonary resuscitation. Crit Care (2013) 17:409. 10.1186/cc11929 23356515 PMC4056022

[6] Goldman S Sutter F Ferdinand F Trace C. Optimizing intraoperative cerebral oxygen delivery using noninvasive cerebral oximetry decreases the incidence of stroke for cardiac surgical patients. Heart Surg Forum (2004) 7:E376–81. 10.1532/HSF98.20041062 15799908

[7] Mohandas BS Jagadeesh AM Vikram SB. Impact of monitoring cerebral oxygen saturation on the outcome of patients undergoing open heart surgery. Ann Card Anaesth. (2013) 16:102–6. 10.4103/0971-9784.109740 23545864

[8] Slater JP Guarino T Stack J Vinod K Bustami RT Brown JM III. Cerebral oxygen desaturation predicts cognitive decline and longer hospital stay after cardiac surgery. Ann Thorac Surg. (2009) 87:36–44; discussion-5. 10.1016/j.athoracsur.2008.08.070 19101265

[9] Edmonds HL Detection and Correction of Brain Oxygen Imbalance: Surgical and Critical Care Applications of the INVOS Cerebral Oximeter. Boulder, CO: Covidien (2014). p. 2–3.

[10] Wong JK Smith TN Pitcher HT Hirose H Cavarocchi NC. Cerebral and lower limb near-infrared spectroscopy in adults on extracorporeal membrane oxygenation. Artif Organs (2012) 36:659–67. 10.1111/j.1525-1594.2012.01496.x 22817780

[11] Cummins RO Chamberlain DA Abramson NS Allen M Baskett PJ Becker L. Recommended guidelines for uniform reporting of data from out-of-hospital cardiac arrest: the Utstein Style. A statement for health professionals from a task force of the American Heart Association, the European Resuscitation Council, the Heart and Stroke Foundation of Canada, and the Australian Resuscitation Council. Circulation (1991) 84:960–75. 1860248 10.1161/01.cir.84.2.960

[12] van Heijst A Liem D Hopman J van Der Staak F Sengers R Oxygenation and hemodynamics in left and right cerebral hemispheres during induction of veno-arterial extracorporeal membrane oxygenation. J Pediatr. (2004) 144:223–8. 10.1016/j.jpeds.2003.11.006 14760266

[13] Liem KD Hopman JC Oeseburg B de Haan AF Festen C Kollee LA. Cerebral oxygenation and hemodynamics during induction of extracorporeal membrane oxygenation as investigated by near infrared spectrophotometry. Pediatrics (1995) 95:555–61. 7700758

[14] Papademetriou MD Tachtsidis I Elliot MJ Hoskote A Elwell CE. Multichannel near infrared spectroscopy indicates regional variations in cerebral autoregulation in infants supported on extracorporeal membrane oxygenation. J Biomed Opt. (2012) 17:067008. 10.1117/1.JBO.17.6.067008 22734786

[15] Fenik JC Rais-Bahrami K. Neonatal cerebral oximetry monitoring during ECMO cannulation. J Perinatol. (2009) 29:376–81. 10.1038/jp.2008.231 19158806

[16] Ejike JC Schenkman KA Seidel K Ramamoorthy C Roberts JS. Cerebral oxygenation in neonatal and pediatric patients during veno-arterial extracorporeal life support. Pediatr Crit Care Med. (2006) 7:154–8. 10.1097/01.PCC.0000200969.65438.83 16446597

[17] Lorusso R Taccone FS Belliato M Delnoij T Zanatta P Cvetkovic M. Brain monitoring in adult and pediatric ECMO patients: the importance of early and late assessments. Minerva Anestesiol. (2017) 83:1061–74. 10.23736/S0375-9393.17.11911-5 28643997

[18] Naguib AN Winch PD Sebastian R Gomez D Guzman L Rice J. The correlation of two cerebral saturation monitors with jugular bulb oxygen saturation in children undergoing cardiopulmonary bypass for congenital heart surgery. J Intensive Care Med. (2017) 32:603–8. 10.1177/0885066616663649 27530512

[19] Schell RM Kern FH Reves JG. The role of continuous jugular venous saturation monitoring during cardiac surgery with cardiopulmonary bypass. Anesth Analg. (1992) 74:627–9. 1567026 10.1213/00000539-199205000-00001

[20] Pozzebon S Ortiz AB Franchi F Cristallini S Belliato M Lheureux O. Cerebral near-infrared spectroscopy in adult patients undergoing veno-arterial extracorporeal membrane oxygenation. Neurocrit Care (2018) 29:94–104. 10.1007/s12028-018-0512-1 29560599

[21] Cove ME. Disrupting differential hypoxia in peripheral veno-arterial extracorporeal membrane oxygenation. Crit Care (2015) 19:280. 10.1186/s13054-015-0997-3 27391473 PMC4511033

[22] Choi JH Kim SW Kim YU Kim SY Kim KS Joo SJ. Application of veno-arterial-venous extracorporeal membrane oxygenation in differential hypoxia. Multidiscip Respir Med. (2014) 9:55. 10.1186/2049-6958-9-55 25389467 PMC4226883

[23] Deegan BM Sorond FA Lipsitz LA Olaighin G Serrador JM. Gender related differences in cerebral autoregulation in older healthy subjects. Conf Proc IEEE Eng Med Biol Soc. (2009) 2009:2859–62. 10.1109/IEMBS.2009.5333604 19964277 PMC2915823

[24] Ghisleni C Bollmann S Biason-Lauber A Poil SS Brandeis D Martin E. Effects of steroid hormones on sex differences in cerebral perfusion. PLoS ONE (2015) 10:e0135827. 10.1371/journal.pone.0135827 26356576 PMC4565711

[25] Zheng Y Villamayor AJ Merritt W Pustavoitau A Latif A Bhambhani R. Continuous cerebral blood flow autoregulation monitoring in patients undergoing liver transplantation. Neurocrit Care (2012) 17:77–84. 10.1007/s12028-012-9721-1 22644887 PMC3748944

[26] Strauss G Hansen BA Kirkegaard P Rasmussen A Hjortrup A Larsen FS. Liver function, cerebral blood flow autoregulation, and hepatic encephalopathy in fulminant hepatic failure. Hepatology (1997) 25:837–9. 10.1002/hep.510250409 9096585

[27] Madsen PL Skak C Rasmussen A Secher NH. Interference of cerebral near-infrared oximetry in patients with icterus. Anesth Analg. (2000) 90:489–93. 10.1097/00000539-200002000-00046 10648345

[28] Buunk G van der Hoeven JG Meinders AE. Prognostic significance of the difference between mixed venous and jugular bulb oxygen saturation in comatose patients resuscitated from a cardiac arrest. Resuscitation (1999) 41:257–62. 10507711 10.1016/s0300-9572(99)00060-x

[29] Watzman HM Kurth CD Montenegro LM Rome J Steven JM Nicolson SC. Arterial and venous contributions to near-infrared cerebral oximetry. Anesthesiology (2000) 93:947–53. 10.1097/00000542-200010000-00012 11020744

[30] Koch S Della-Morte D Dave KR Sacco RL Perez-Pinzon MA. Biomarkers for ischemic preconditioning: finding the responders. J Cereb Blood Flow Metab. (2014) 34:933–41. 10.1038/jcbfm.2014.42 24643082 PMC4050240

[31] Wang W Yu XD Mo X Zhang HB Zhu DM Limb ischemic preconditioning attenuates cerebral ischemic injury in arat model. Perfusion (2014) 29:210–8. 10.1177/0267659113503681 24002779

[32] Steppan J Hogue CW Jr. Cerebral and tissue oximetry. Best Pract Res Clin Anaesthesiol. (2014) 28:429–39. 10.1016/j.bpa.2014.09.002 25480772 PMC4258229

